# Severe combined cardiac and neuromuscular toxicity from immune checkpoint blockade: an institutional case series

**DOI:** 10.1186/s40959-020-00076-6

**Published:** 2020-09-23

**Authors:** Puja Arora, Laura Talamo, Patrick Dillon, Ryan D. Gentzler, Trish Millard, Michael Salerno, Craig L. Slingluff, Elizabeth M. Gaughan

**Affiliations:** 1 University Hospitals Siedman Cancer Center, Westlake, OH USA; 2 Department of Medicine, Division of Hematology, Duke University, Durham, NC USA; 3 Department of Medicine, Division of Hematology and Medical Oncology, University of Virginia, PO Box 800716, Charlottesville, VA 22908 USA; 4 Department of Medicine, Division of Cardiovascular Medicine, Noninvasive Cardiovascular Imaging, Nuclear Medicine, University of Virginia, Charlottesville, VA USA; 5 Department of Surgery, Division of Surgical Oncology, University of Virginia, Charlottesville, VA USA

**Keywords:** Combination immunotherapy, Immune related adverse events, Myocarditis, Myasthenia gravis, Myositis

## Abstract

**Background:**

Immune checkpoint inhibition is part of standard systemic management for many advanced malignancies. Toxicities from this treatment approach are unpredictable, though usually reversible with management per established guidelines. Some patients suffer major morbidity and treatment-related mortality from these agents in an unpredictable manner. Cardiac and neurologic complications are rare, but can result in serious clinical consequences.

**Methods:**

We describe the presentation, management, and outcomes of eight sequential cases of combined cardiac and neurologic toxicities resulting in severe illness and demonstrating lack of rapid response to immunosuppression.

**Results:**

Our cohort consisted of six males and two females with an average age of 73.5 years (61–89 years). There were four patients with melanoma, and one patient each with urothelial carcinoma, renal cell carcinoma, breast cancer, and non-small cell lung cancer. Four patients received combination immunotherapy and four patients received monotherapy. The median time to presentation from treatment initiation was 27 days (11–132 days). All patients had a cardiovascular and neurologic toxicity, and most had hepatitis and myositis. The cardiac signs and symptoms were the prominent initial features of the clinical presentation. Each patient was managed by a multidisciplinary team and received a range of immunosuppressive agents. All patients died as a consequence of the immune related adverse events.

**Conclusions:**

The evaluation of patients with cardiac adverse events from immunotherapy, should include assessment of overlapping toxicities such as myasthenia gravis and myositis. Providers should be aware of the potential for an extended duration of disability and slow improvement for certain toxicities as these expectations may factor prominently in goals of care decisions.

## Background

Immune checkpoint inhibitors (ICI), including antibodies against cytotoxic T-lymphocyte associated antigen-4 (CTLA-4), programmed cell death-1 (PD-1), and programmed-death-ligand-1 (PD-L1), function to help the body overcome potential barriers to the recognition and elimination of cancer by the host immune system. There are numerous monotherapy and combination indications for these drugs in the management of advanced cancer. Approvals in the adjuvant setting have further expanded the population of patients with both potential benefit and toxicity from treatment [1, 2]. Patient exposure to ICI will continue to increase, as there are several new agents in development.

Toxicities from ICI therapy, termed immune related adverse events (irAEs), can occur with any ICI agent, and can manifest any time during or after therapy. Constitutional symptoms, dermatologic, gastrointestinal, hepatic, and endocrine toxicities are the most common irAEs and management strategies are outlined in published guidelines and expert reviews [3–6]. Less common irAEs, such as cardiac and neurologic toxicities, have emerged with increasing utilization. Despite the low frequency, these complications are often serious, with a variety of clinical profiles described [6]. There are reports of fulminant fatal cardiac toxicity, electrical dysfunction, acute coronary syndrome, pericarditis, and acute systolic heart failure occurring after ICI therapy [7–13]. Defining the frequency and patterns of cardiac toxicity from ICI therapy is an area of active investigation [14]. The spectrum of neurologic toxicity can range from non-specific symptoms, such as headache and weakness, to classic findings of well-defined neurologic disorders. A 2019 pharmacovigilance study reported an increased incidence of myasthenia gravis (MG), encephalitis, peripheral neuropathy and meningitis with use of ICI therapy [15].

Despite the low frequency of these toxicities, our single academic institution managed eight patient cases since April 2016 of severe clinical illness resulting from concurrent cardiac and neurologic irAEs, all resulting in fatal outcomes.

## Methods

Patients were identified through clinical care at admission to the University of Virginia (UVA) Health System. After Institutional Review Board review, we collected clinical and pathologic data related to malignancy diagnosis and treatment and past medical history. We evaluated the presentation of each subject, including clinical descriptions in progress notes, results from laboratory testing, cardiovascular analysis, and cross-sectional imaging. We outlined the immunosuppressive regimens used in each case and the recorded outcomes. Descriptive statistics were reported.

### Case 1

**Fig. 1:**
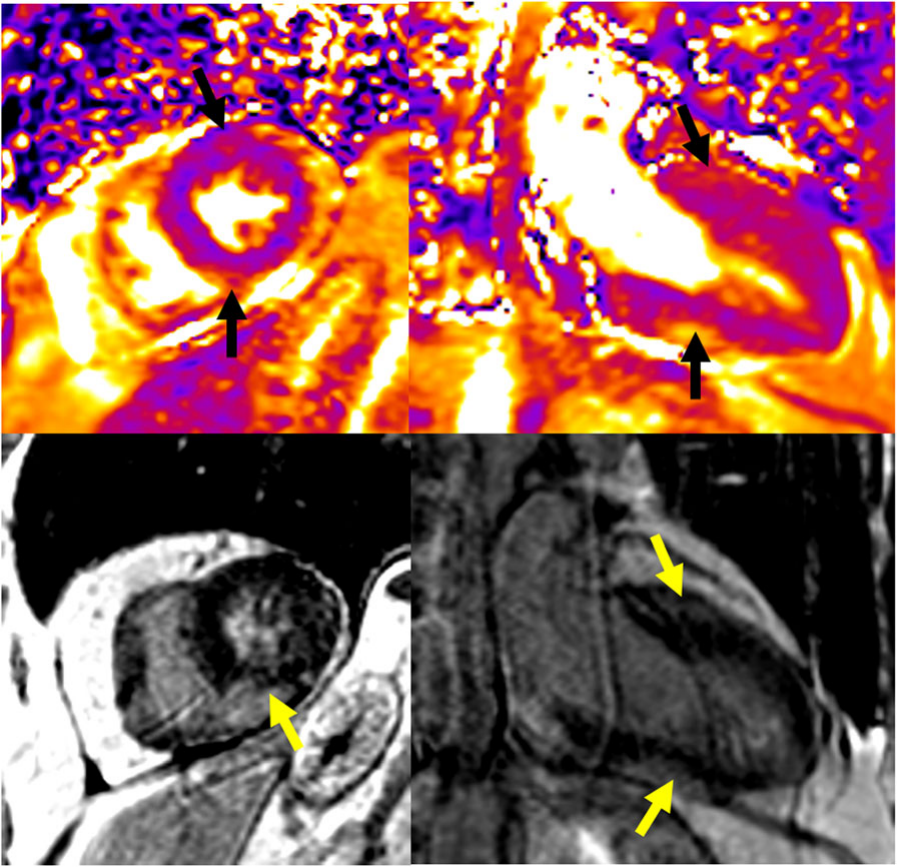
**Cardiac MRI from Case 1**: T2 maps (top row) demonstrate patchy focal areas of myocardial edema in the anterior wall and inferior wall (black arrows). Late gadolinium enhancement (LGE) images (bottom row) demonstrate patchy focal areas of scar (yellow arrows). These findings are consistent with myocarditis

**Fig. 2:**
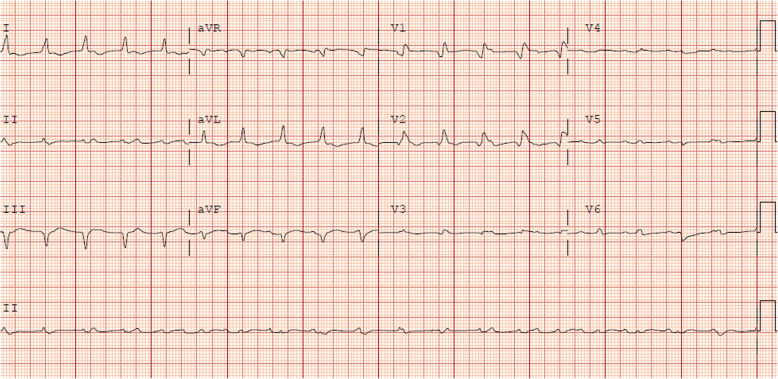
**Electrocardiogram for Case 1**. Taken on the final hospital day in the setting of clinical decline and three hours prior to final cardiac arrest. Junctional tachycardia, abnormal R-wave progression, prolonged QTc (532 msec)

The patient was a 70-year-old male with metastatic melanoma treated with combination ipilimumab and nivolumab. He presented on cycle 1, day 12, with palpitations, double vision, right ptosis and pre-syncope. Initial troponin was 3.65 ng/mL and creatinine kinase (CK) was 3549 U/L. Transthoracic echocardiogram (TTE) showed normal left and right ventricular (LV/RV) function and electrocardiogram (ECG) revealed sinus tachycardia, right bundle branch block, and premature atrial contractions. The patient was started on IV steroids at 1 mg/kg for presumed checkpoint inhibitor induced myocarditis. Cardiac magnetic resonance imaging (CMR) revealed evidence of myocardial edema and late gadolinium enhancement in the subepicardial mid-inferior wall consistent with acute myocarditis (Fig. 1). The patient developed progressive cardiac electrical abnormalities and the troponin continued to rise to 29.69 ng/mL. Anti-thymocyte globulin (ATG) was started and the steroid dose was increased to one-gram methylprednisolone with his declining clinical status. The patient had cardiac arrest and responded to CPR. Telemetry demonstrated bradycardia with complete heart block and pacing was initiated. Patient had short periods of clinical stabilization but no sustained improvement from a cardiac or neurologic standpoint with aggressive supportive care and immunosuppression. On hospital day 5, mycophenolate mofetil (MMF) and cyclophosphamide were added to the regimen but by the next day, the patient had significant global weakness with progressive bilateral ptosis and dyspnea. Plasmapheresis was started for the clinically diagnosis of Myasthenia gravis (MG) and anti-acetylcholine-receptor (AChR) antibody was positive. Repeat TTE demonstrated worsening LV and RV function with LVEF of 40–45% and troponin rose to 90.58 ng/mL. He required intubation for respiratory failure, developed progressive and refractory electrical abnormalities and ultimately died after unsuccessful resuscitation efforts (Fig. 2).

### Case 2

**Fig. 3:**
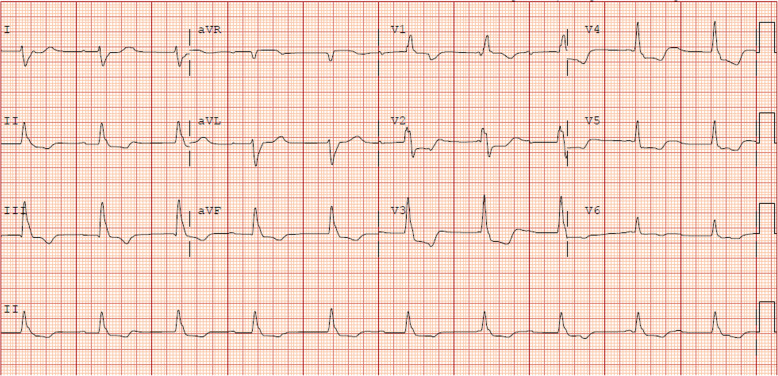
**Presenting Electrocardiogram for Case 2**. Complete atrioventricular block with wide QRS complex

he patient was a 79-year-old male with metastatic melanoma treated with pembrolizumab. He presented with blurred vision, diplopia, fatigue, lower extremity weakness and diffuse pain. The patient was found to be in complete heart block with an initial troponin of 16.45 ng/mL and CK of 11,953 U/L, as well as hepatitis (ALT 395 U/L, AST 710 U/L) (Fig. 3). Bedside TTE showed a LVEF of 60–65%. His exam was notable for ophthalmoplegia, bilateral fatigable ptosis, and diffuse weakness with muscle pain during strength testing. The patient was started on IV steroids at 1 mg/kg initially, but the dose was increased to 1 g daily on hospital day (HD) 1 along with initiation of ATG and MMF with rising biomarkers. Intravenous cyclophosphamide was started on hospital day 3. Cardiac biomarkers ultimately decreased with this therapy, to a nadir of troponin 0.34 ng/mL and CK 247 U/L. He completed the planned courses of intravenous steroid and ATG and was transitioned to oral prednisone. A permanent pacemaker was placed, and he had no further cardiac events during his illness. Intravenous Immunoglobulin (IVIg) was initiated for the clinical diagnosis of MG, but there was no improvement in generalized weakness or ophthalmoplegia. His anti-AChR antibody testing was negative. His course was further complicated by an upper gastrointestinal bleed due to erosive duodenopathy and a pulmonary embolism. Patient opted to transition to hospice care due to lack of significant clinical improvement.

### Case 3

**Fig. 4:**
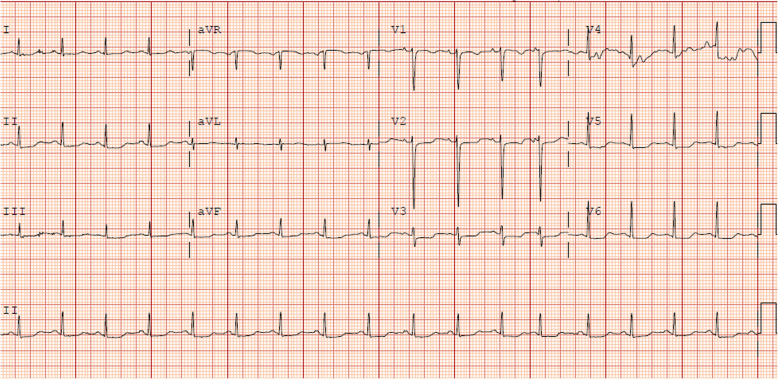
**Electrocardiogram from Patient 3**. Taken in the setting of chest pain, dizziness and dyspnea. Prolonged QTc 519 msec (baseline 437 msec)

The patient was a 61-year-old female with metastatic breast cancer treated with combination durvalumab and tremelimumab on trial. The patient presented for dose two of therapy with several days of right ptosis and was found to have hepatitis (AST 720 U/L, ALT 327 U/L), myositis (CK 8082 U/L) and elevated troponin (1.03 ng/mL). She was started on IV steroids at 2 mg/kg and was admitted to the hospital. TTE was performed and demonstrated LVEF of 60–65% and CMR did not show evidence of myocarditis. Urgent brain MRI was negative for brain metastases or leptomeningeal disease. For a clinical diagnosis of MG, she received pyridostigmine with symptomatic improvement. Her anti-AChR antibody testing was negative. The IV steroids induced steady improvement in transaminases, and CK. She experienced episodes of chest pain, dizziness, dyspnea, and developed premature atrial contractions and QTc prolongation on ECG (Fig. 4). Her troponin initially improved to 0.86 ng/mL with therapy, but on it rose again to a maximum of 1.24 ng/mL. MMF was added and the troponin steadily improved to 0.65 ng/mL at discharge. She returned to the hospital within 2 days of discharge with chest pressure and dizziness. Her troponin was 0.39 ng/dL and her transaminases were also trending downward. Her sodium, normal at discharge, was 119 mmol/L and the patient was transitioned back to IV steroids. For a diagnosis of syndrome of inappropriate antidiuretic hormone (SIADH), she required hypertonic saline. She declined quickly with the development of hypercapnic respiratory failure and was transitioned to comfort measures.

### Case 4

The patient was a 69-year-old male with metastatic urothelial carcinoma treated with pembrolizumab. The patient presented with diffuse body pain and weakness on cycle 5, day 2, of pembrolizumab. His troponin was elevated at 1.42 ng/mL, and he was managed for non-ST-elevation myocardial infarction (NSTEMI). His troponin continued to climb to a peak of 23 ng/mL over the first 24 h. CK peak was 372 U/L at arrival but then normalized with supportive care. TTE demonstrated mild concentric hypertrophy with EF of 40–45% and CMR was not pursued due to chronic kidney disease. IV steroids were initiated at 1 mg/kg initially along with MMF given concern for ICI-induced myocarditis. Cardiac catheterization did not show evidence of acute coronary artery obstruction. A course of ATG was initiated and the steroids were increased to one gram of methylprednisolone daily for rising cardiac biomarkers. The patient clinically improved and troponin levels peaked at 58.99 ng/mL on HD 6 and then decreased to 20.21 ng/mL by the end of the clinical course. CK remained low and no clinically significant cardiac electrical abnormalities were identified. The presenting global weakness, disconjugate gaze and diffuse body pain did not improve. On HD 4, the patient developed altered mental status due to multiple cerebrovascular accidents resulting in left hemiparesis. Further workup for the etiology of the strokes was unrevealing, with vasculitis or cholesterol-emboli from the catheterization as the leading considerations. The patient opted to transition to comfort measures.

### Case 5

The patient was a 67-year-old female with metastatic melanoma treated with combination ipilimumab and nivolumab. She presented on cycle 1 day 5 with diffuse body weakness, dyspnea, and dysphagia and was found to have hepatitis (ALT 563 U/L, AST 353 /L) and a troponin of 1.8 ng/mL. Once hospitalized, she soon developed hypercapnic respiratory failure and was intubated. TTE demonstrated preserved EF, pro-B-type NP was 1370 pg/mL, and CK was 7244 IU/L. She was considered unstable for cardiac catheterization. Patient was started on IV steroids at 2 mg/kg and MMF. The troponin rose to 12.02 ng/mL, while CK and transaminases trended down. With failure to clinically improve, she was transferred to our institution and the steroids were increased to methylprednisolone 1 g per day and ATG was added. Repeat TTE demonstrated an EF of 60–65%. Her troponin peaked at 18.42 ng/dL and then declined. CMR was performed 8 days after starting immunosuppression with improving cardiac biomarkers (CK 231, Troponin 1.86) and did not demonstrate imaging evidence of myocarditis. Immunosuppression was tapered and cardiac and hepatic biomarkers showed steady improvement. The patient remained ventilator-dependent due to respiratory muscle weakness from a clinical diagnosis of MG. Her anti-AChR antibody testing was positive. Plasmapheresis was initiated but failed to impact her clinical status and she was transitioned to comfort measures.

### Case 6

The patient was an 83-year-old male with melanoma treated with nivolumab in the adjuvant setting. One month after his first dose, he developed marked fatigue, weakness, chest pain and orthopnea. His troponin was 0.78 ng/mL and he was diagnosed with pericarditis and medically managed with colchicine and naproxen. The next day, he presented again with chest tightness, dysphagia, and progressive left eye ptosis. TTE showed LVEF of 55% and treatment for pericarditis was continued. On examination, he had proximal muscle weakness, areflexia and ocular muscle abnormalities. He had both hepatitis (ALT 166 U/L, AST 510 U/L) and an elevated CK (2886 U/L). MRI of the brain was unremarkable. The patient was started on IV methylprednisolone at 1 mg/kg and plasmapheresis was started for a clinical diagnosis of MG. His anti-AChR antibody testing was negative. He required intubation for hypercapnic respiratory failure. Over the course of the next several days his troponin, CK and liver function tests all improved, but he had persistent respiratory compromise with inability to reduce ventilatory support. The patient was transitioned to comfort measures.

### Case 7

The patient was a 70-year-old male with metastatic kidney cancer treated with ipilimumab and nivolumab in combination. He presented on cycle 1 day 21 with generalized weakness and fatigue. He was identified to have a CK of 11,000 U/L and troponin of 36 ng/mL. The patient was treated with IV steroids and supportive care with stabilization of clinical status and biochemical improvement. He was discharged to a rehabilitation facility and transitioned to oral prednisone for a taper. The patient clinically deteriorated with progressive dysphagia, globus sensation, dyspnea and weakness on prednisone at 60 mg (approximately 0.5 mg/kg). He was admitted with a clinical diagnosis of ICI-induced MG and plasmapheresis was started. His anti-AChR antibody testing was negative. The patient required intubation for respiratory failure and then the steroids were increased to methylprednisolone 1 g per day. His troponin level peak value was 0.28 ng/mL on this hospitalization. His echocardiogram demonstrated a preserved LVEF of 60–65% and no major rhythm abnormalities were identified. He developed upper gastrointestinal bleeding from immune-related gastritis and Infliximab was given. The patient had persistent respiratory failure and was transitioned to comfort measures.

### Case 8

The patient is an 89-year-old male with non-small cell lung cancer treated with pembrolizumab. The patient presented 10 days after dose 2 with disconjugate gaze, dysphagia, blurred vision and imbalance and was found to have hepatitis (ALT 243 U/L, AST 416 U/L). For a clinical diagnosis of MG, he was started on IV steroids at 1 mg/kg. The anti-AChR testing was negative. He had an elevated troponin to 1.66 ng/mL at presentation that peaked at 2.34 ng/mL, favored clinically to be checkpoint-inhibitor-induced myocarditis. TTE was technically limited and cardiac MRI had evidence of a prior infarct with no clear evidence of myocarditis and an ejection fraction of 47%. He showed multiple episodes of non-sustained ventricular tachycardia and developed high-degree atrioventricular block. For elevated CK to 3843 U/L, he underwent EMG showing myopathic changes expected with myositis. Liver function tests and CK improved with steroids but patient had persistent weakness, dysphagia and blurred vision. The patient and family opted to pursue comfort measures.

## Results

### Clinical and oncologic data

Eight successive cases of combined neurologic and cardiac toxicities presented to the UVA Health System, including four patients who received oncologic care outside of our institution and were transferred for toxicity management (Table 1). This group included six males and two females, all Caucasian, with an average age of 73.5 years (range, 61–89 years). There were five diseases represented, including four patients with melanoma, and one patient each with urothelial carcinoma, breast carcinoma, renal cell carcinoma, and non-small cell lung cancer.

**Table 1:** Demographics and Patient Information

Case	Age	Sex	Disease	ICI Therapy	Time to Presentation from ICI Initiation	Baseline Medical History
1	70	Male	Melanoma	Ipilimumab/Nivolumab	11 Days	Hypertension Colon Cancer
2	79	Male	Melanoma	Pembrolizumab	26 Days	CLL
3	61	Female	Breast Cancer	Durvalumab/Tremelimumab	28 Days	None
4	69	Male	Urothelial Carcinoma	Pembrolizumab	132 Days	CKD, Hypertension, Hyperlipidemia
						Type 2 DM
						CAD
5	67	Female	Melanoma	Ipilimumab/Nivolumab	14 Days	None
6	83	Male	Melanoma	Nivolumab, Adjuvant	31 Days	Hypertension,
						Hyperlipidemia
						Atrial fibrillation
7	70	Male	Renal Cell Carcinoma	Ipilimumab/Nivolumab	21 Days	Hypertension, CKD, Atrial Fibrillation
8	89	Male	Non-Small Cell Lung Carcinoma	Pembrolizumab	32 Days	Hypertension, Hyperlipidemia, CAD, CKD, Type 2 DM

Baseline data, disease and treatment information for each patient. *CLL* chronic lymphocytic leukemia, *CAD* coronary artery disease, *Type 2 DM* type 2 diabetes mellitus, *CKD* chronic kidney disease

Four patients received combination anti-CTLA4 and anti-PD1, including two patients with melanoma, one with breast cancer and one patient with renal cell carcinoma (Table 1). Four patients received anti-PD-1 monotherapy, including one patient being treated in the adjuvant setting for melanoma. Five patients had baseline cardiovascular comorbidities, including coronary artery disease, hypertension, type 2 diabetes mellitus, and atrial fibrillation (Table 1). One patient had a concurrent diagnosis of chronic lymphocytic leukemia requiring chemotherapy and one patient had a remote history of resected colon cancer. None had prior autoimmune disease.

### Toxicity presentation

The patients presented at a median of 27 days (range, 11–132 days) from the initiation of ICI therapy. All cases had a cardiovascular toxicity recognized early in the acute presentation (Table 2). Seven cases had a clinical syndrome consistent with myocarditis, including three with high-degree atrioventricular conduction block. The eighth patient was diagnosed with pericarditis. All patients were diagnosed with presumed checkpoint-inhibitor myocarditis based on clinical features (history, examination, biomarkers, electrocardiogram and cardiac imaging) and each patient was assessed by a Cardiology specialist. There were no cardiac biopsies or autopsies performed for histologic assessment.

**Table 2:** Range of toxicities for each case

Case	Cardiovascular Toxicity	Neurologic/Ocular Toxicity	Myositis	Hepatitis	Respiratory Failure	Other
1	Myocarditis Complete Heart Block	Myasthenia Gravis	+	+	+	–
2	Myocarditis, Complete Heart Block, Pulmonary Embolism	Myasthenia Gravis	+	+	–	Gastritis
3	Myocarditis	Myasthenia Gravis	+	+	–	SIADH
4	Myocarditis	Stroke	–	–	–	–
5	Myocarditis	Myasthenia Gravis	+	+	+	–
6	Pericarditis	Myasthenia Gravis	+	+	+	–
7	Myocarditis	Myasthenia Gravis	+	+	+	Gastritis
8	Myocarditis, Complete Heart Block	Myasthenia Gravis	+	+	–	–

Each case had multiple overlapping toxicities after presenting with primarily a cardiac symptom. *SIADH* syndrome of inappropriate anti-diuretic hormone

All cases had a neurologic toxicity, primarily MG (Table 2). The eighth patient had multiple strokes. Of the cases of MG, five had ophthalmoplegia and four developed respiratory failure requiring intubation. Most patients had concurrent myositis, diagnosed by elevated creatine kinase (CK) and clinical symptoms. There were no muscle biopsies performed but one patient had characteristic myositis findings on EMG. All patients were evaluated by Neurology specialists.

Two patients developed severe gastritis/duodenitis with upper gastrointestinal bleeding, favored immune in origin. Gastric biopsy from case seven showed findings consistent with checkpoint inhibitor gastritis. One patient ultimately died of hypercapnic respiratory failure in the setting of complications from the syndrome of inappropriate anti-diuretic hormone (SIADH) and the relationship with the checkpoint inhibitor therapy is not known.

### Toxicity treatment

Multiple immunosuppressive agents were used in each case. All patients were treated with steroids with starting doses between 1 and 2 mg/kg and additional agents were added in a stepwise approach determined by the patient’s clinical status and response. Steroids were continued for the duration of the clinical course. Guidelines from the American Society of Clinical Oncology (ASCO) and the National Comprehensive Cancer Network (NCCN®) were followed and expert opinion was sought within and outside of our academic medical center. Four patients ultimately received five-day courses of one-gram intravenous methylprednisolone. Several of these patients presented within months of each other, which impacted the immunosuppression dosing and agent timing and selection. Four patients (50%) received anti-thymocyte globulin, four (50%) received mycophenolate mofetil, and two patients (25%) had cyclophosphamide added to the regimen for lymphodepletion in the setting of persistent clinical illness. For the MG, all patients were treated symptomatically with pyridostigmine. Four patients (50%) underwent plasmapheresis and one received intravenous immunoglobulin. One patient received infliximab for irAE management. The early immunosuppressant choices were targeted at the cardiac abnormalities and the antibody depleting therapies for MG tended to start in the first several days of admission.

### Outcomes

All patients died as a result of the immunotherapy toxicity (Table 3). The average time between the last treatment and death was 32.5 days (range, 17–67 days). One patient died rapidly in the hospital despite maximal interventions. The other seven opted for withdrawal of aggressive care with transition to comfort measures. In six cases (75%), the cardiac components improved with down-trending biomarkers, maintenance of stable ejection fraction, and lack of significant electrical instability apart from the pacemaker required for cases with complete heart block. One patient died of progressive cardiac failure on a fulminant course. One patient was showing developing cardiac electrical changes including high-degree atrioventricular block at the time that care was transitioned to comfort measures.

**Table 3:** Patient Outcome

Case	Cardiac Improvement	Neurologic Improvement	Myositis Improvement	Hepatitis Improvement	Respiratory Improvement	Cancer Response	Withdrawal of Care/Hospice Decision	Time from last treatment to Death
1	–	–	+	+	–	SD	–	17 Days
2	+	–	+	+	NA	PR	+	21 Days
3	+	+	+	+	NA	NR	+	41 Days
4	+	–	NA	NA	NA	SD	+	17 Days
5	+	–	+	+	–	SD	+	34 Days
6	+	–	+	+	–	NA	+	42 Days
7	+	–	+	+	–	NR	+	67 Days
8	–	–	–	–	NA	NR	+	21 Days

Outcome of immunosuppression on toxicity course by organ system and overall course. For organ toxicities “+” = improved, “- “= did not improve. *NA* Not applicable, *SD* stable disease, *PR* Partial response, *NR* Not reported

Only one case had notable improvement from the neurologic toxicity and the generalized weakness was a prominent aspect of the toxicity course for each patient. None of the patients with respiratory failure requiring mechanical ventilation recovered sufficiently to be removed from the ventilator. As each of these patients and/or families made the decision to withdraw care, the trajectory and potential neurologic and respiratory recovery with prolonged support is not known.

Six of the seven (86%) cases with myositis showed significant improvement in CK levels with immunosuppression. Six of the seven (86%) of the cases with hepatitis showed significant improvement in transaminase levels with immunosuppression. The seventh patient in both of these groups discontinued interventions prior to evidence of improvement.

Four of the eight (50%) cases had cross-sectional imaging done during the disease course that allowed assessment of cancer response to therapy. Of these four, one patient showed a partial response to therapy and three had stable disease.

## Discussion

Toxicities from ICI are variable in onset, presentation, and severity. In current experience, there are no patient or tumor characteristics that reliably predict irAEs. Our cases included men and women, ages from 61 to 83 years, with varied cancers and ICI-treatments represented (Table 1). All patients had metastatic disease, including one patient that had undergone melanoma resection to no evidence of disease and was receiving therapy in the adjuvant setting. Three patients had minimal comorbid conditions. Each patient had evidence of a cardiovascular insult and a neurologic insult, and most had concurrent hepatitis and myositis. Toxicities tended to onset early following initiation of ICI therapy, often after the first dose. In this cohort, the cardiovascular signs and symptoms were visible early in the presentation and were the focus of initial diagnostics and immunosuppressive interventions. The neurologic insults, however, proved to be the most symptomatic and the primary determinants of the clinical course.

Cardiac toxicity from ICI therapy was a rare immune-related adverse event in the early ICI trials, but this complication is becoming increasingly recognized and reported [16, 17]. In one series of over 20,000 patients treated with nivolumab, ipilimumab or combined ipilimumab and nivolumab, cardiovascular irAEs were rare (0.09%) [7]. In addition, Mahmood identified an overall incidence of myocarditis of 1.14% from a multicenter registry [18]. With increasing use of ICI agents, the spectrum of associated cardiovascular toxicity is expanding. In this cohort, clinical profiles included fulminant myocarditis, complete heart block, QTc prolongation, pulmonary embolism, and pericarditis. Each patient also had several concurrent irAEs, many with overlapping myocarditis, myositis, and Myasthenia gravis. In 2018, Moslehi et al. reported on 101 cases of severe myocarditis from ICI therapy and noted that in 42% of cases, there was a concurrent severe irAE, most commonly myositis and Myasthenia [19]. Mortality reported from these cases of myocarditis ranged from 36% for patients treated with anti-PD-1 or anti-PD-L1 antibodies to 67% for patients treated with combination anti-CTLA-4 and anti-PD1/PD-L1 therapy.

In 2016, Johnson reported two cases of fulminant myocarditis in patients receiving combination ipilimumab and nivolumab for advanced melanoma [8]. Both patients received aggressive immunosuppression and supportive care but quickly died from the irAE. The clinical course for two of our cases followed a similar fulminant pattern with early onset symptoms after ICI initiation, electrical instability, and rapid clinical deterioration. One patient died and one patient survived the initial cardiac instability. For most of our patients, the cardiac insults proved manageable in the short term with aggressive supportive care, including device placement and immunosuppression, evidenced by preserved ejection fraction, lack of major electrical dysfunction, and improving cardiac biomarkers. As each patient died, we can make no comments regarding the long-term cardiac outcomes.

Seven of our cases had a clinical syndrome consistent with Myasthenia gravis. MG occurs in approximately 0.15% of patients treated with anti-PD1 therapy [20]. This is an antibody-mediated disorder of the post-synaptic membrane at the neuromuscular junction, and, in its severe form, can impact respiratory muscle function [21, 22]. The diagnosis of MG is usually clinical but can be supported by the presence of autoantibodies, typically against the acetylcholine receptor [21]. In each of our cases, the MG diagnosis was made on clinical grounds by a Neurologist. Antibody testing was done on each patient, but results took several days to return, and treatment decisions were required without the results. There are several other autoantibodies less commonly implicated in the development of MG, including antibodies against the voltage-gated potassium channel Kv1.4, which are associated with cardiac involvement of the Myasthenia [23, 24]. Treatment for MG often includes immunosuppression and antibody-depleting therapy, such as plasmapheresis and/or intravenous immunoglobulin. While corticosteroids are the mainstay of management for irAEs, there is a concern about worsening of weakness early in the course of MG with initiation of steroids. Early multidisciplinary input is invaluable to management of these complex toxicities.

As with all irAEs, ICI-induced MG presents with a spectrum of severity with some patients only developing limited symptoms that respond to immunosuppression and pyridostigmine [25, 26]. Overall, ICI-induced MG tends to have a more aggressive clinical course than non-ICI-related disease and patients often show signs of myositis and myocarditis [20, 27]. The triad of MG, myositis and myocarditis has been reported in case reports of ICI-treated patients with a variety of cancers [20, 28–34]. Suzuki et al. reported the results of a safety database in Japan including outcomes of 10,277 patients treated with either single-agent nivolumab or ipilimumab [27]. Twelve patients developed MG, with early onset of symptoms with rapid clinical deterioration. Ten of these 12 patients had elevated CK levels, four with confirmed concurrent myositis, and three with concurrent myocarditis. Six of these patients developed a myasthenic crisis including five patients that required respiratory support for a median duration of 54 days (range 10–128 days). The authors report that the patients with severe myasthenia had a slow recovery with improvement in muscle strength occurring over 4–8 weeks. In a review by Kao et al., the rates of respiratory failure with ICI-induced MG were as high as 50% of reported cases, and most patients presented with elevated levels of CK [20]. In our cohort, four patients out of seven with a clinical syndrome of MG required intubation for respiratory failure and each had an elevated CK.

Clinically significant objective and subjective weakness was a prominent issue throughout the clinical course for most of our cases. In the setting of advanced cancer, the marked clinical deterioration and prolonged duration of symptoms were central components of the care decisions. Seven of the patients opted to transition to best supportive care due to ongoing setbacks and/or severe weakness after 2–4 weeks of aggressive care. Despite objective improvements in measurable laboratory and clinical parameters, and supportive interventions for mobility and strength, the patient-reported weakness failed to meaningfully improve. In contrast to the expected rapid improvement with immunosuppression often seen in management of most irAEs, the clinical course for our cases was strikingly different with improvement in objective measures, but minimal overall clinical change for these patients. Providers should be aware of the potential long duration of illness and debility from some severe irAEs in order to counsel patients and family members, as these expectations will likely factor into care decisions.

We remain without clinical, pathologic, or pharmacologic predictive features for the development of the combination of cardiac and neurologic irAEs and therefore, oncologists and patients balance the risk of rare serious toxicity with the substantial potential benefit of the treatments. The heterogeneity of our cases reflects the general experience that these toxicities can impact any patient on any checkpoint inhibitor and providers need to be aware of the potential for rapid onset of toxicity and clinical deterioration. The potential for long and slow recovery is an important expectation to set with patients experiencing certain combined irAEs. Providers are encouraged to evaluate for overlapping irAEs particularly in the setting of severe presentations and a multidisciplinary management approach should be strongly considered. The morbidity and mortality from these rare irAEs will take on additional significance as the ICI agents move further into the adjuvant setting.

## Conclusions

In the evaluation of patients with cardiac adverse events from immunotherapy, providers should evaluate for overlapping toxicities such as myasthenia gravis and myositis. Providers should be aware of the potential for an extended duration of disability and slow improvement for certain toxicities as these expectations may factor prominently in goals of care decisions.

## Data Availability

Data sharing is not applicable to this article as no datasets were generated or analyzed during the current study.

## Abbreviations

ICI: Immune checkpoint inhibition
CTLA-4: Cytotoxic T-lymphocyte associated protein-4
PD-1: Programmed cell death protein 1
PD-L1: Programmed death-ligand 1
irAEs: Immune-related adverse events
MG: Myasthenia gravis
UVA: University of Virginia
CK: Creatine Kinase
SIADH: Syndrome of inappropriate anti-diuretic hormone
CLL: Chronic lymphocytic leukemia
CAD: Coronary artery disease
CKD: Chronic kidney disease
Type 2 DM: Type 2 diabetes mellitus
ATG: Anti-thymocyte globulin
MMF: Mycophenolate mofetil
Cyclophos: Cyclophosphamide
IVIg: Intravenous immunoglobulin
